# Supplementation with MitoTEMPO before cryopreservation improves sperm quality and fertility potential of Piedmontese beef bull semen

**DOI:** 10.3389/fvets.2024.1376057

**Published:** 2024-05-15

**Authors:** Ahmed R. Elkhawagah, Alessandro Ricci, Alessia Bertero, Mariagrazia Lucia Poletto, Tiziana Nervo, Gian Guido Donato, Leila Vincenti, Nicola Antonio Martino

**Affiliations:** ^1^Theriogenology Department, Faculty of Veterinary Medicine, Benha University, Banha, Egypt; ^2^Department of Veterinary Sciences, University of Turin, Grugliasco, Italy; ^3^Department of Biosciences, Biotechnology and Environment, University of Bari Aldo Moro, Bari, Italy

**Keywords:** bull semen, cryopreservation, MitoTEMPO, sperm quality, DNA, mitochondrial activity, *in vitro* embryo production

## Abstract

The purpose of this study was to improve the quality of frozen–thawed Piedmontese bull semen by incorporating MitoTEMPO (MT) in extended semen before cryopreservation. Semen was collected from 4 fertile bulls, using an artificial vagina, once weekly for 6 consecutive weeks. Semen samples were pooled, diluted with Bullxcell^®^ extender, and supplemented with different concentrations of MT (0 as control, 5, 10, 20, 40, and 80 μM) before cooling, equilibration, and freezing procedures. The frozen–thawed semen was assessed for motility, vitality, acrosome intactness, plasma membrane integrity, DNA integrity, apoptosis, mitochondrial membrane potential, intracellular ROS level and *in vitro* fertilizing capability. The results showed that MT at concentrations of 10, 20, and 40 μM improved the total, progressive, and rapid motility directly after thawing while, at the highest tested concentration (80 μM), it decreased the progressive and rapid motility after 1, 2, and 3 h of incubation. The sperm kinetics including STR and LIN were noticeably increased at concentrations of 10, 20, and 40 μM directly after thawing (0 h), whereas the MT effect was variable on the other sperm kinetics during the different incubation periods. MitoTEMPO improved the sperm vitality at all tested concentrations, while the acrosomal and DNA integrity were improved at 20 μM and the mitochondrial membrane potentials was increased at 80 μM. The cleavage and blastocyst formation rates were significantly increased by using semen treated with 20 μM MT compared with controls. These findings suggest a potential use of MT mainly at a concentration of 20 μM as an additive in the cryopreservation media of bull semen to improve sperm quality.

## 1 Introduction

The Piedmontese breed is the most numerous Italian beef breed, characterized by muscular hypertrophy due to a specific mutation in the myostatin gene (1). It is highly specialized for beef production due to the double muscling characteristic that exerts positive effects on carcass conformation, dressing percentage, and meat quality (2).

Artificial insemination is a breeding method implemented worldwide that accounts for an increasing proportion of cattle reproduction (3). Semen cryopreservation is an important factor for artificial insemination (4), as it allows for the use of cattle genetic resources by making semen of genetically superior bulls available worldwide (5). Therefore, advances in bull semen cryopreservation and sperm fertility are critical economic traits for breeding programs (6). Cryopreservation induces several biophysical and biochemical changes in the sperm membrane that markedly decrease the sperm fertility potential (7). Cryopreservation has been associated with excessive production of reactive oxygen species (ROS), which is the major reason for reduced frozen semen quality (4, 8). Furthermore, sperm cells are highly susceptible to lipid peroxidation (LPO) as they contain high levels of polyunsaturated fatty acids (PUFA), which detrimentally affect sperm motility and membrane integrity (9).

Mitochondria is one of the primary sources of sperm reactive oxygen species (ROS) (10). ROS are produced by electron leakage from the electron transport chain (ETC), which is accepted by molecules of oxygen resulting in the production of O_2_^−^ (11, 12). Furthermore, ROS is produced by the mitochondrial apoptotic pathway, which is triggered when the phosphoinositide signaling pathway is disrupted (13). Additionally, the presence of a high level of PUFA in mitochondrial membranes, which serve as preferential ROS substrates, promotes lipid peroxidation and the production of lipid aldehydes. These bind to ETC proteins covalently, increasing mitochondrial ROS production (14, 15).

Antioxidant supplementation of extenders has been used to protect sperm from oxidative stress and improve sperm quality after thawing (4). Depending on their mechanism of action, antioxidants are classified as enzymatic or non-enzymatic substances that scavenge free radicals (16, 17). Non-enzymatic antioxidants include endogenous compounds such as amino acids, glutathione, and coenzyme Q10 as well as exogenous compounds such as polyphenols, carotenoids, vitamin E, and vitamin C. Enzymatic antioxidants include catalase (CAT), superoxide dismutase (SOD), glutathione transferase (GST), glutathione peroxidase (GPx), thioredoxins (TRX), and peroxiredoxins (PRDXs) (18). Mito-Tempo is a mitochondria-targeted superoxide dismutase mimetic that scavenges superoxide molecules by converting them into hydrogen peroxide or oxygen and then detoxifies them to oxygen and water by catalase or glutathione peroxidase (19, 20). Therefore, using mitochondria-targeted antioxidants could help to preserve the activity and fertility potential of cryopreserved sperm cells (21). Mitochondria-targeted antioxidant (MitoTEMPO, MT) is a novel antioxidant and a powerful cell-permeable ROS scavenger that protects cells from oxidative stress in a variety of conditions by scavenging superoxide anion in the catalytic cycle (22–24). MitoTEMPO contains the antioxidant piperidine nitroxide (Tempo) and the lipophilic cation, triphenylphosphonium (TPP+) and accumulates within mitochondria (25). The addition of MT to semen extender has a sperm quality protective effect in frozen–thawed human (26, 27), rooster (28), buffalo (29) and buck (30) sperm as well as chilled ram semen (24).

The influence of MT on the fertility parameters of cryopreserved bull semen has not been recorded. Therefore, the present study aims to investigate the effect of MT supplementation to semen extender before cryopreservation on post-thawing sperm motility, vitality, mitochondrial membrane potential (MMP), membrane functionality, DNA integrity, intracellular ROS level, as well as sperm *in vitro* fertilizing capability.

## 2 Materials and methods

**Figure 1:**
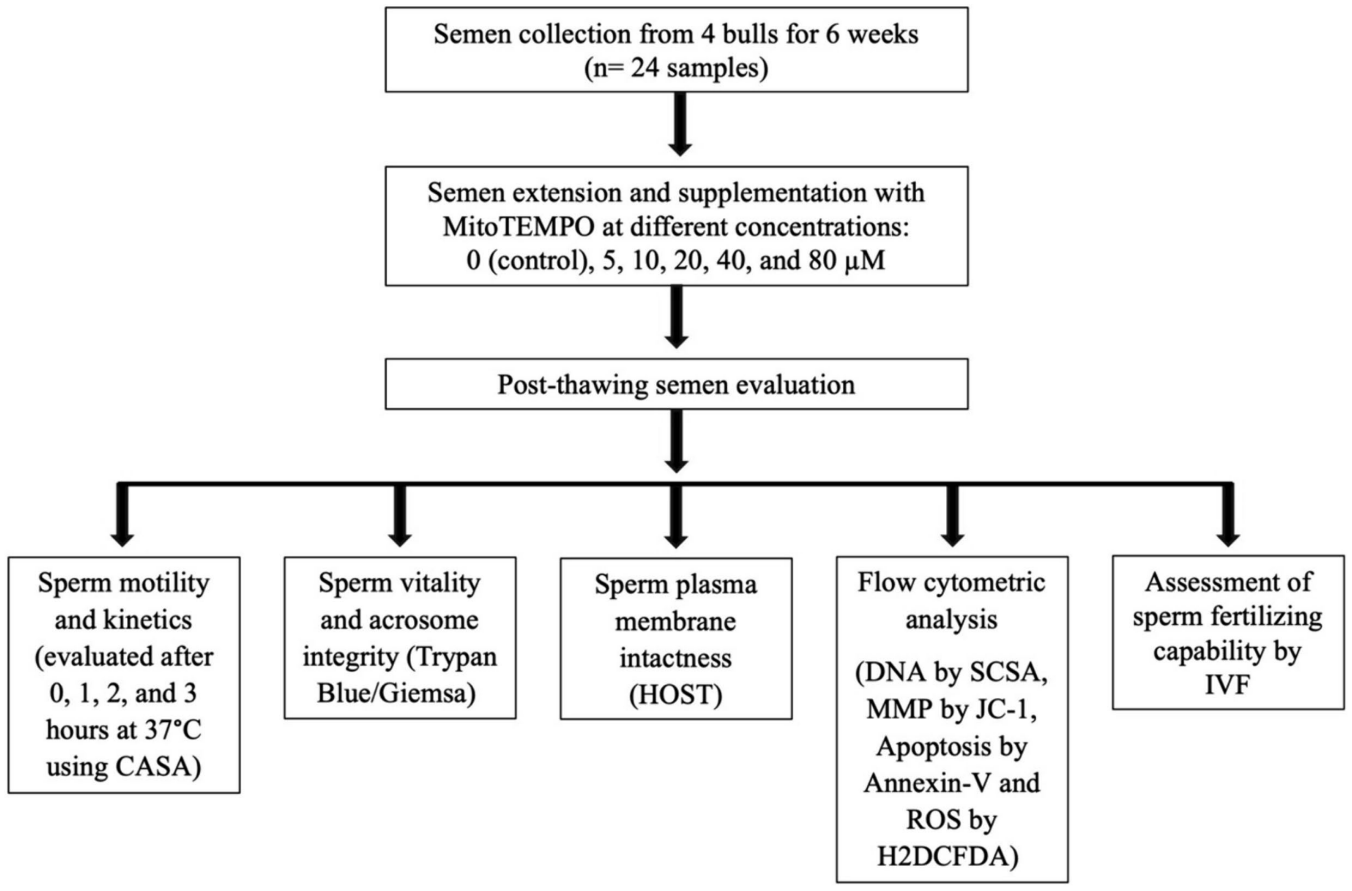
Schematic form of the experimental design. CASA, computer-assisted semen analysis; HOST, hypoosmotic swelling test; MMP, mitochondrial membrane potential; SCSA, sperm chromatin structural assay; ROS, reactive oxygen species; H2DCFDA, 2′,7′-Dichlorodihydrofluorescein diacetate; IVF, *in vitro* fertilization.

The experimental design flowchart is presented in Figure 1.

### 2.1 Ethics approval statement

No experimental animals were used in this study. All procedures were performed in accordance with DPR 27/1/1992 (Animal Protection Regulations of Italy) in compliance with European Community Regulation 86/609.

### 2.2 Semen collection and extension

For semen collection, four Piedmontese bulls (1.5–2 years old) of proven fertility at the ANABORAPI center in Carrù, Piemonte region, Italy, were used. From each bull, semen was collected once weekly for 6 consecutive weeks (6 ejaculates/bull with a total of 24 ejaculates) using a bovine artificial vagina adjusted at 45°C. Samples with concentration ≥ 800 × 10^6^/mL and motility ≥70% were used. Pooled semen samples were extended with Bullxcell^®^ extender (IMV, France) following the manufacturer’s instructions, and supplemented with MitoTEMPO (MT, SML0737, Sigma-Aldrich, Italy) at different concentrations: 0 (control), 5, 10, 20, 40, and 80 μM. The MT solution was prepared by dissolving the content of the MT vial in 1 mL TALP medium (as detailed below) followed by storage as aliquots of 70 μL at-80°C until use. The doses used in this study were determined depending on the results reported in previous publications in different species including humans (26, 27), ram (24), boar (31), rooster (28), buffalo (29), and buck (30). Extended semen containing 30 × 10^6^ spermatozoa/mL was cooled to 5°C and then packed into 0.5 mL polyvinyl straws (Minitube, Germany) followed by equilibration for 4 h (h) and freezing using a controlled-rate freezer (SY-LAB Gerate GmbH, Neupurkersdorf, Austria). Semen freezing was performed at five predetermined rates: extended semen was cooled at a rate of-4°C/min from +4°C to-9°C. In the range of −9°C to −25°C, the freezing rate was −50°C/min. From −25°C to −100°C, the freezing rate dropped to −35°C/min, −20°C/min from −100°C to −144°C, and − 4°C/min from −144°C to −150°C. After reaching −150°C, the straws were dipped in liquid nitrogen (−196°C). At least after 24 hin liquid nitrogen, straws were thawed (*N* = 4/trial/treatment) at 37°C for 40 s for the various assessments.

### 2.3 Semen evaluation

#### 2.3.1 Assessment of sperm motility and kinetics

The thawed sperm motility and kinetics were evaluated after different periods of incubation (0, 1, 2, and 3 h) at 37°C using the computer-assisted sperm analyzer (CASA; Hamilton Thorne, Inc., Beverly, MA, United States). In a prewarmed Mackler chamber, 10 μL semen specimens were loaded according to the manufacturer’s instructions and evaluated with a bull semen-specific setup; 30 frames were obtained at a 60 Hz frame rate, with a minimum cell size of 8 pixels and a minimum contrast of 40. The VAP and VSL cutoff times were 15 μ/s and 4.4 μ/s, respectively. The speed standards were set as fast; >80 μm/s, medium; >60 μm/s, slow; >20 μm/s and static. The motility (total, progressive, and rapid, %) and velocities such as Straight linear velocity μm/s (VSL), Curvilinear velocity μm/s (VCL), Beat cross-frequency Hz (BCF), Average path velocity μm/s (VAP), Amplitude of lateral head displacement, μm (ALH), Straightness (STR, [VSL/VAP] × 100) and Linearity (LIN, [VSL/VCL] × 100) parameters, were assessed in eight randomly selected fields.

#### 2.3.2 Assessment of sperm vitality and acrosomal integrity

**Figure 2:**
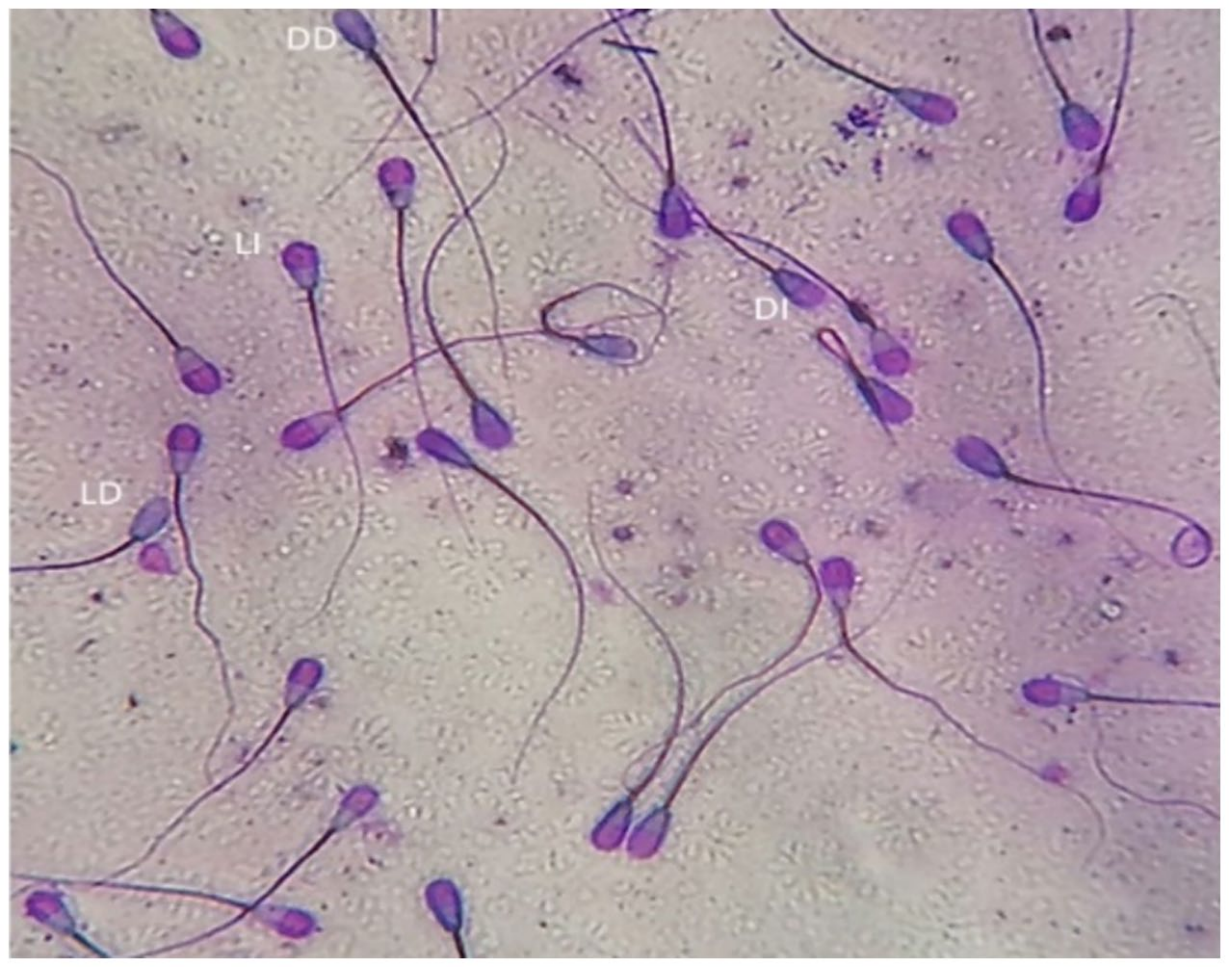
Bull spermatozoa stained with Trypan blue/Giemsa stain. Four types of stained sperm were identified: LI, live with intact acrosome, LD, live with damaged acrosome, DI, dead with intact acrosome, DD, dead with damaged acrosome.

The dual staining technique (Trypan Blue/Giemsa) according to Boccia et al. (32) was used to evaluate sperm vitality and acrosomal status. Equal volumes of semen and trypan blue (0.27%) were mixed and smeared on a glass slide and allowed to dry before being fixed for 4 min in 37% formaldehyde with neutral red. The fixed smears were stained overnight with Giemsa (7.5%), after which, slides were washed with distilled water and allowed to dry before being microscopically examined (Advanced Automated Research Microscope System, Nikon Eclipse E200, phase contrast at 40x and 100x magnifications, United States). Four different types of stained sperm were identified: live with intact acrosome, live with damaged acrosome, dead with intact acrosome, and dead with damaged acrosome (Figure 2). Two-hundred sperm cells were evaluated for each experimental group.

#### 2.3.3 Assessment of sperm plasma membrane intactness

**Figure 3:**
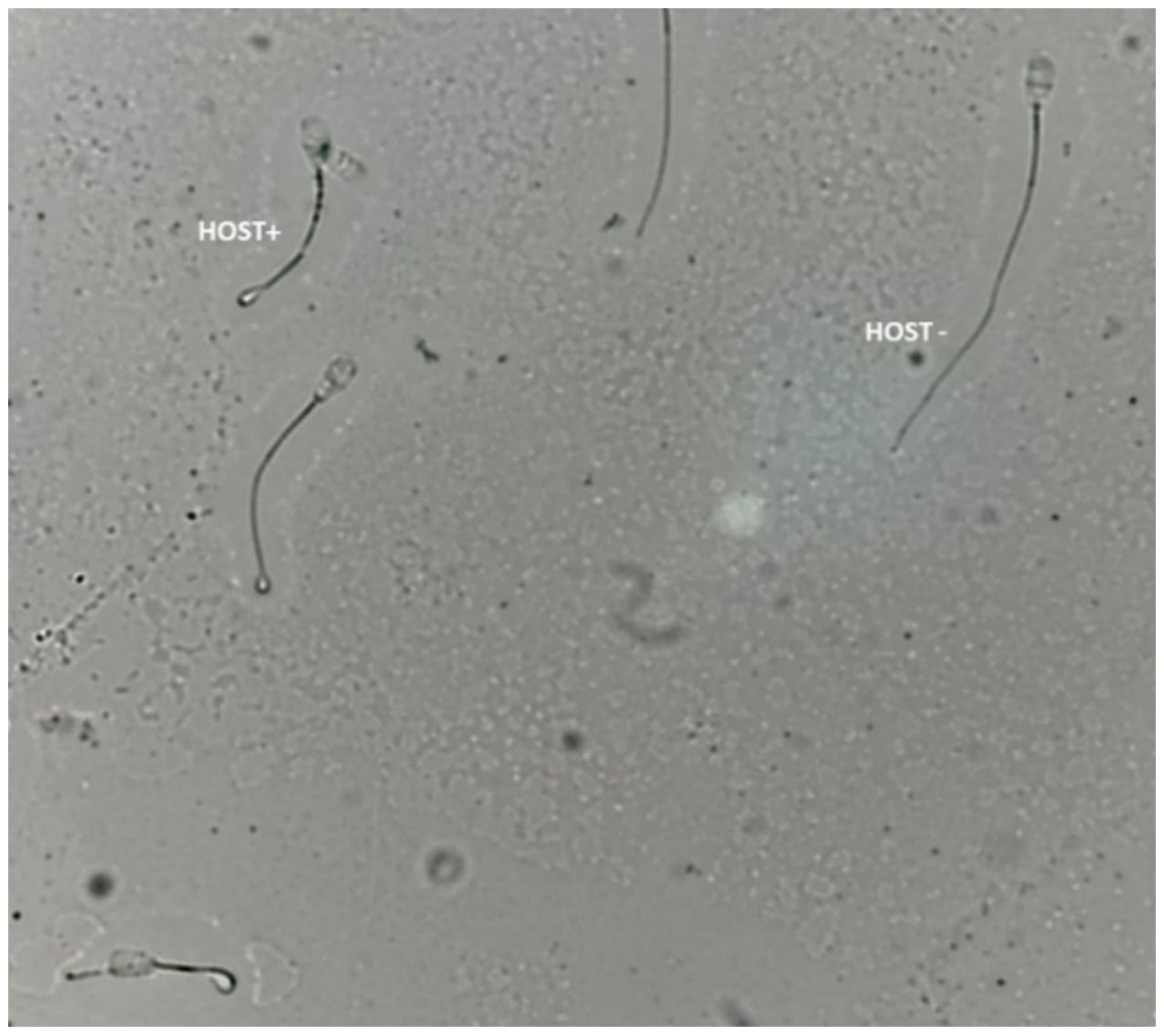
Bull sperm plasma membrane integrity assessed by the hypoosmotic swelling test (HOST). HOST+; sperm with an intact plasma membrane, HOST-; sperm with the damaged plasma membrane.

According to Akhter et al. (33), the hypo-osmotic swelling test (HOST) was used to evaluate the intactness of the sperm plasma membrane. A 50 μL semen specimen mixed with 500 μL of prewarmed HOST solution (0.735 g sodium citrate and 1.351 g fructose dissolved in 100 mL of distilled water with an osmotic pressure of approximately 190 mOsm/kg) was incubated for 40 min at 37°C. Using a phase contrast microscope (400X), at least 200 spermatozoa/slide were assessed for the percentage of cells with intact plasma membrane (curled/swollen tails; HOST positive, Figure 3).

#### 2.3.4 Flow cytometric analysis

A FacsStar Plus flow cytometer (Becton Dickinson Immunochemistry, San Jose, CA, United States) equipped with standard optics and an air-cooled argon laser at 488 nm and 15 mW was used to assess the integrity of sperm DNA (SCSA), mitochondrial membrane potential (JC-1), apoptosis (Annexin-V/PI binding assay), and intracellular ROS level (H2DCFDA). Using CellQuest^®^ software (BD Biosciences, San Jose, CA, United States) and a flow rate of 200 events/s, a total number of 10.000 gated events were analyzed per sample.

##### 2.3.4.1 Assessment of sperm DNA integrity using SCSA

**Figure 4:**
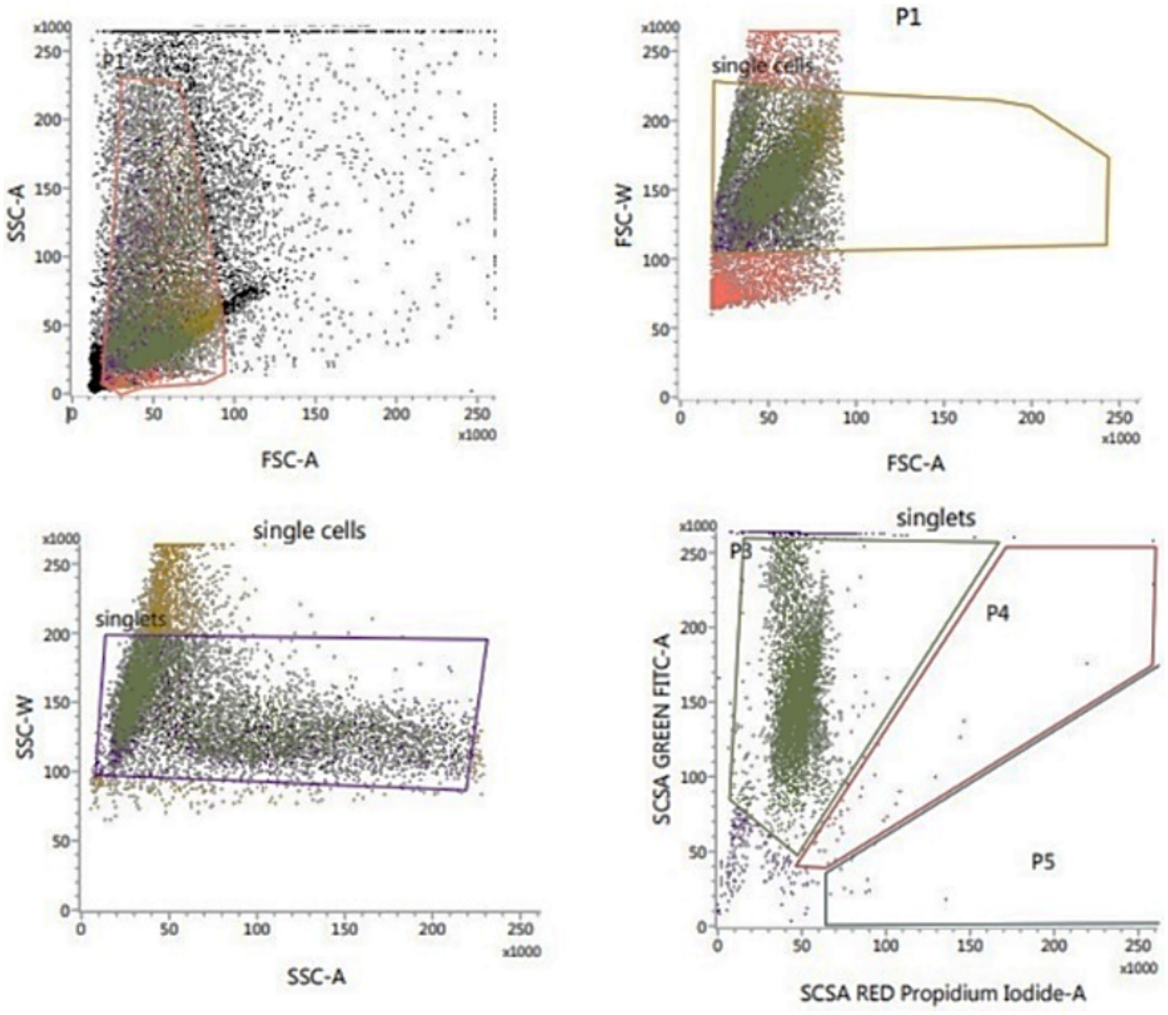
Flow cytometric evaluation of sperm DNA integrity using SCSA.

The sperm DNA integrity was assessed flow cytometrically using sperm chromatin structure assay (SCSA; Figure 4) according to Evenson and Jost (34). The thawed sperm were washed in phosphate buffered saline (PBS) by centrifugation at 500 g for 10 min. The sperm pellets were diluted to a final concentration of 2 × 106 sperm/mL with TNE buffer (0.01 M Tris–Cl, 0.15 M NaCl, 1 mM EDTA, disodium pH 7.4) followed by the addition of 400 μL of acid detergent solution (0.08 N HCl, 0.15 M NaCl, 0.1% (w/v) Triton X-100, pH 1.2). After 30 s, semen was stained with 1,200 μL of Acridine Orange (AO, Sigma-Aldrich, United States) staining solution containing 600 μL AO (6 mg/mL) diluted in 100 mL staining buffer (0.037 M citric acid, 0.126 M Na2HPO4, 1.1 mM EDTA disodium, 0.15 M NaCl, pH 6.0) followed by the Flowcytometric evaluation. The cells’ red (single-stranded DNA) and green (double-stranded DNA) fluorescence were recorded. The fluorescence was measured using FL1 (530/15 nm) and FL3 (650 nm) filters. The percentage of sperm with double-stranded DNA was recorded.

##### 2.3.4.2 Assessment of sperm mitochondrial activity

**Figure 5:**
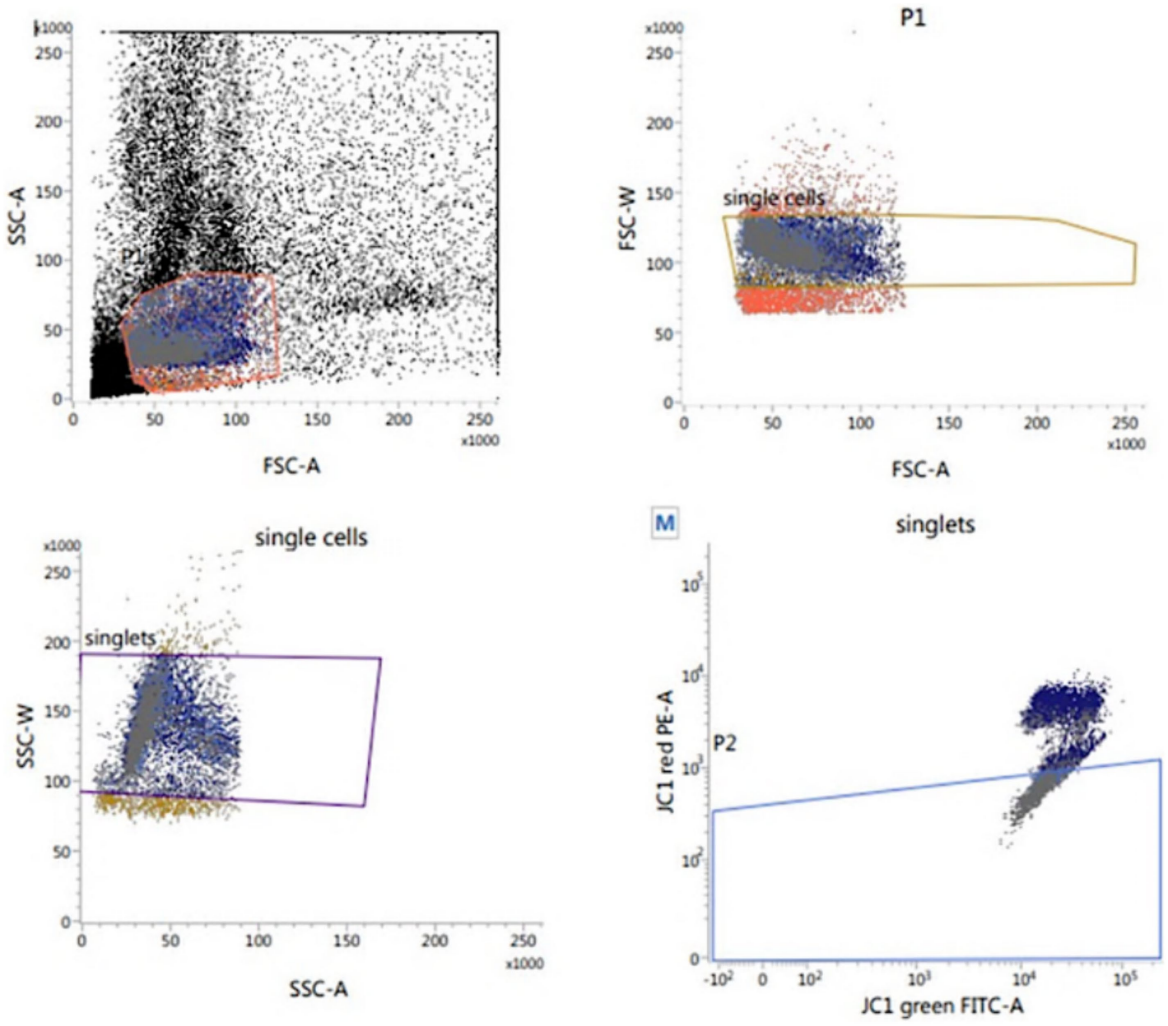
Flow cytometric evaluation of sperm mitochondrial membrane potential (MMP) using JC-1.

According to Martinez-Pastor et al. (35), the sperm mitochondrial activity was assessed using the lipophilic cation JC-1 (MitoProbe™ JC-1 Assay Kit for Flow Cytometry; M34152), Fisher Scientific – Scheepsbouwersweg 1b – Postbus 4 – 1120 AA Landsmeer; Figure 5. Thawed sperm (1 × 10^6^) were washed in 1 mL PBS by centrifugation at 500 g for 10 min. After centrifugation, the sperm pellets were resuspended in 1 mL PBS and supplemented with 10 μL of JC-1 (JC-1200 μM in DMSO), and incubated for 15 to 30 min at 37°C, 5% CO2. At the end of incubation, semen was centrifuged in 2 mL PBS, followed by resuspension in 500 μL PBS and assessed Flow Cytometrically. With a 488 nm laser, JC-1 was excited and by using the emission filters of 535 nm and 595 nm, the cells with green (JC-1 monomers) and orange (JC-1 aggregates) fluorescence were quantified. Using the frequency plots of FL1 (green) and FL2 (orange), the percentage of sperm stained green and orange was determined. The percentage of orange-stained sperm was recorded as cells of high mitochondrial membrane potential (HMMP) cells.

##### 2.3.4.3 Evaluation of sperm for apoptosis (Annexin-V/PI-binding assay)

**Figure 6:**
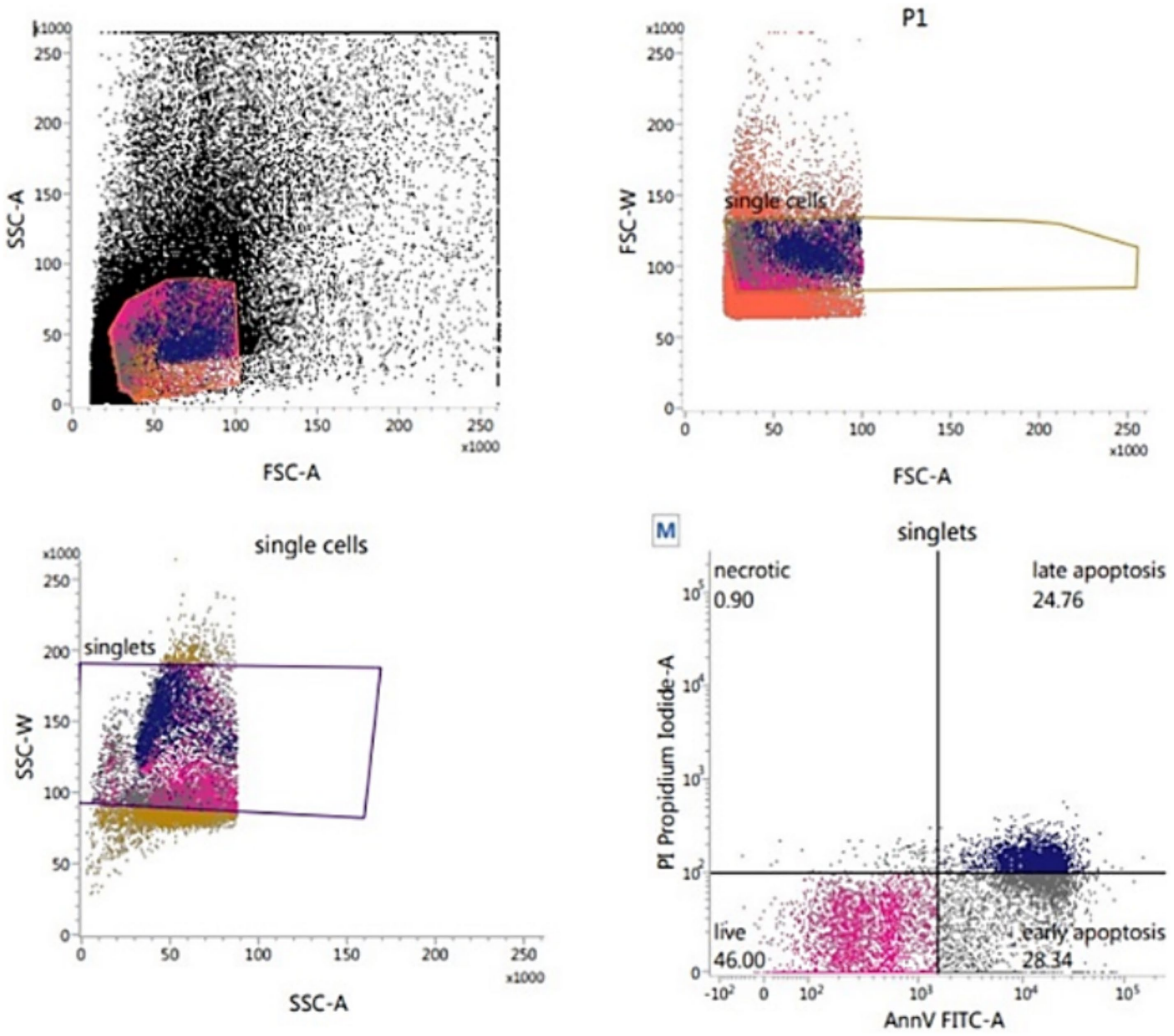
Flow cytometric evaluation of sperm apoptosis using Annexin-V apoptosis kit.

The sperm plasma membrane integrity and apoptosis were assessed by using Alexa Fluor 488 Annexin-V Apoptosis Kit (INVITROGEN – V13245) and Propidium Iodide (Figure 6) according to Anzar et al. (36). Thawed sperm (1 × 10^6^) were washed in 1 mL PBS by centrifugation at 500 g for 10 min. Sperm pellets were resuspended in 100 μL of Annexin-V-binding buffer (10 mM HEPES, 140 mM NaCl, 2.5 mM CaCl2, pH 7.4), and supplemented with 1 μL of PI (100 μg/mL) and 5 μL of Annexin-V before being gently mixed and incubated in the dark for 15 min at room temperature. At the end of incubation, 400 μL of Annexin-V-binding buffer was added before flow cytometric evaluation which was conducted as soon as possible. By using the annexin V/PI binding assay, the orthogonal light scatter (SSC), forward light scatter (FSC), FITC fluorescence (FL1), and PI fluorescence (FL3) were assessed. To eliminate particles and restrict the analysis only to spermatozoa, an acquisition gate was used in the FSC/SSC two-dimensional histogram. The percentage of apoptotic (annexin V+ and PI+), early apoptotic (annexin V+ and PI¯), necrotic (annexin V¯ and PI+), and viable (annexin V¯ and PI¯) spermatozoa was recorded.

##### 2.3.4.4 Assessment of sperm intracellular ROS levels

**Figure 7:**
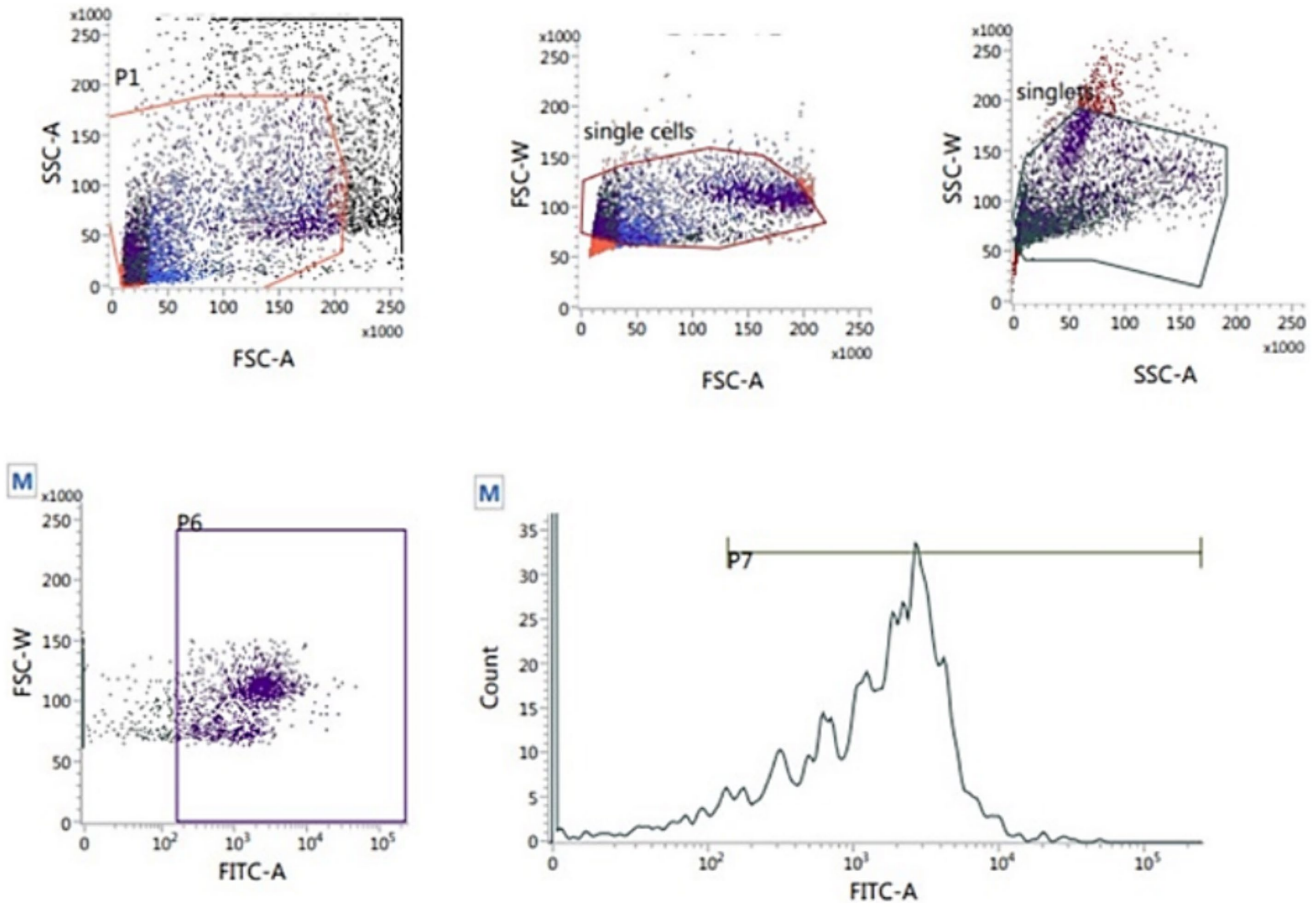
Flow cytometric evaluation of sperm ROS using T2′,7′-dichlorodihydrofluorescein diacetate (H2DCFDA) staining kit.

The 2′,7′-dichlorodihydrofluorescein diacetate (H2DCFDA, Abcam Cellular ROS Assay Kit, ab113851) was used to measure the sperm intracellular ROS level according to Gallo et al. (37). Thawed sperm (1 × 10^6^) were washed in 1 mL PBS by centrifugation at 500 g for 10 min. The sperm pellets were resuspended in 500 μL freshly prepared stain (10 μM H2DCFDA) and incubated at 37°C for 30 min in the dark. After incubation, the samples were analyzed flow cytometrically. The percentage of sperm ROS was recorded on the fluorescence intensity of the emission spectrum that was measured from 500 to 560 nm (typically FL1) with an excitation wavelength of 488 nm (Figure 7).

### 2.4 Assessment of sperm fertilizing capability

Based on the effects of MT on sperm quality parameters, the fertilizing capacity of thawed semen treated with 20 μM MitoTEMPO was evaluated *in vitro* using bovine oocytes and compared with control in the absence of MT. Briefly, Piedmontese bovine ovaries were collected from the slaughterhouse (Manzo Carni, Cuneo, Italy), washed, and transported in warm saline solution (38°C) to the laboratory within 2 h. After rinsing with warm saline, follicles 2–8 mm in diameter were aspirated using a 10 mL syringe and an 18-gauge needle under the stereo microscope (ZEISS^®^, Carl Zeiss Suzhou Co., Ltd. Germany). Sediments of the aspirated follicular fluid were transferred to a 100 mm dish, and cumulus-oocyte complexes (COCs) with multiple layers of intact compact cumuli were selected and washed three times in TCM-199 HEPES (supplemented with 10% FCS) and three times in the *in vitro* maturation medium. The oocyte *in vitro* maturation was performed as reported in Elkhawagah et al. (38) in TCM-199 Earle’s Salt Medium supplemented with 10% FCS, 5 μg/mL follicle-stimulating hormone (FSH; Folltropin-V^®^, Bioniche Animal Health.), 5 μg/mL luteinizing hormone (LH; Lutropin-V^®^, Bioniche Animal Health, Belleville, ON, Canada.), 1 mg/mL 17β-estradiol, 0.2 mM sodium pyruvate, and 10 μg/mL gentamycin (38). The oocytes were cultured in wells containing 400 μL IVM medium covered with paraffin oil and incubated at 38.5°C in 5% CO_2_. After 24 h, thawed semen was washed by centrifugation at 300 g for 30 min in a Percoll discontinuous gradient by adding 1 mL Percoll 90% under 1 mL Percoll 45% and semen was layered on the top in a 15 mL tube. At the end of the IVM culture, the oocytes underwent *in vitro* fertilization (IVF) in Tyrode’s-albumin-lactate-pyruvate (TALP) medium containing 2 mM penicillamine, 1 mM hypo- taurine, 250 mM adrenaline, 20 mg mL^−1^ heparin, 114 mM NaCl, 3.2 mM KCl, 0.4 mM NaH_2_PO_4_, 10 mM sodium lactate, 25 mM NaHCO_3_, 0.5 mM MgCl_2_ x 6H_2_0, 2.0 mM CaCl_2_ x 2H_2_O, 6 mg mL-^1^ BSA, 5 mL mL^−1^ gentamicin and 0.2 mM sodium pyruvate (39). Specifically, the semen (with or without MT treatment) was added to IVF droplets containing the oocytes (10 μL semen/droplet) at the final concentration of 10 × 10^6^ spermatozoa/ml. After gametes co-incubation at 38.5°C for 20 h, cumulus cells were removed, and zygotes were washed and cultured for 7 days at 38.5°C with 5% CO_2_ and 5% O_2_ in synthetic oviductal fluid (SOF) (40): 1.1 M NaCl, 72 mM KCl, 12 mM KH_2_PO_4_, 7.4 mM MgSO_4_, 50 mM D, L-lactate, 250 mM NaHCO_3_, 260 mM phenol red, 100 mM sodium pyruvate, 178 mM CaCl_2_ 2H_2_O, 125 mM HEPES sodium salt, 30.8 mM glutamine, 500 mM glycine, 84.2 mM alanine, 100x minimum essential medium (MEM) non-essential amino acids, 50x basal medium Eagle (BME) amin oacid solution, 2.8 mM myoinositol, 340 mM trisodium citrate, 2% FCS, 0.005gmL^−1^ BSA, 0.2 mM sodium pyruvate and 5mLmL^−1^ gentamicin. At the end of incubation, embryos were fixed with 2% paraformaldehyde, mounted on a glass slide, and stained with Hoechst 33258. Embryos were evaluated for cleavage and blastocyst formation rates using a Nikon Eclipse TE 2000-S fluorescence microscope (Nikon^®^, Nikon Solutions Co., Ltd., Shinagawa, Japan) fitted with a B2A (346 nm excitation/460 nm emission) filter.

### 2.5 Statistical analysis

Semen data were analyzed and presented as mean ± SEM using SPSS vers. 25 software (IBM^®^, Rome, Italy). The general linear model (GLM) multivariate with Duncan test was used to make multiple comparisons of the means. The fertilizing potential was assessed using the Chi-squared test, considering the cleavage rate and blastocyst formation rate. Statistical significance was set for *p* < 0.05.

## 3 Results

### 3.1 Effect of MitoTEMPO on motility parameters

The mean values of sperm motility parameters evaluated by CASA analysis are shown in Table 1. Sperm samples treated with MT at 10, 20, and 40 μM showed increased total sperm motility (*p* < 0.01) when assessed directly after thawing. Similarly, sperm samples treated with MT at 20 and 40 μM, after 1 h of incubation, and those treated with MT at 5 and 20 μM, after 2 h of incubation, showed a significant increase in total sperm motility. On the contrary, after 3 h of incubation, a marked decrease in the total sperm motility was found when high MT concentrations (20, 40, and 80 μM) were used (*p* < 0.01), compared to controls. Interestingly, the progressive and rapid motilities were improved in all MT-treated samples when assessed directly after thawing (*p* < 0.01), with the best values recorded in the MT 20 μM group. However, a marked decrease in these parameters was recorded, after 1 and 2 h of incubation, in samples treated with MT at 80 μM, compared to the other experimental groups (*p* < 0.01). Compared to the controls, a significant decrease in the rapid sperm motility was recorded with the lowest concentration of MT (5 μM) after 3 h of incubation (*p* < 0.05).

**Table 1:** Effect of MitoTEMPO (MT) on motility parameters (Mean ± SEM) of cryopreserved Piedmontese bull semen after different incubation periods.

Parameter	Group	Post-thawing	1 h	2 h	3 h
Total motility (%)	Control	75.92 ± 1.19^a^	63.44 ± 1.98^ab^	42.94 ± 2.13^ab^	37.15 ± 1.25^a^
	MT 5 μM	77.69 ± 1.66^abc^	65.88 ± 1.75^abc^	48.77 ± 2.36^b^	33.73 ± 1.36^ab^
	MT 10 μM	81.42 ± 0.90^c^	68.98 ± 2.19^bc^	45.67 ± 1.84^ab^	34.77 ± 1.35^ab^
	MT 20 μM	81.19 ± 1.15^c^	70.71 ± 1.81^c^	48.40 ± 1.72^b^	32.75 ± 1.65^b^
	MT 40 μM	80.58 ± 1.01^bc^	69.60 ± 1.80^c^	42.96 ± 2.40^ab^	30.79 ± 0.92^b^
	MT 80 μM	77.00 ± 1.50^ab^	61.06 ± 1.98^a^	41.00 ± 1.60^a^	31.54 ± 1.31^b^
	Sig.	0.01	0.01	0.05	0.01
Progressive motility (%)	Control	39.06 ± 1.18^a^	31.73 ± 1.35^a^	13.63 ± 1.32^a^	2.94 ± 0.46
	MT 5 μM	41.85 ± 0.95^b^	31.46 ± 1.22^a^	13.40 ± 1.06^a^	2.06 ± 0.36
	MT 10 μM	44.46 ± 0.77^bc^	32.63 ± 1.21^a^	13.67 ± 0.89^a^	2.90 ± 0.37
	MT 20 μM	46.31 ± 0.93^c^	31.00 ± 0.83^a^	10.77 ± 0.88^a^	2.67 ± 0.49
	MT 40 μM	43.71 ± 0.80^bc^	30.90 ± 1.27^a^	10.94 ± 0.89^a^	2.15 ± 0.28
	MT 80 μM	41.71 ± 0.82^b^	25.96 ± 1.08^b^	6.83 ± 0.62^b^	2.08 ± 0.33
	Sig.	0.01	0.01	0.01	NS
Rapid motility (%)	Control	57.60 ± 1.29^a^	42.56 ± 1.81^a^	22.69 ± 1.70^a^	11.79 ± 0.80^a^
	MT 5 μM	60.94 ± 1.35^b^	41.44 ± 1.57^a^	22.04 ± 1.71^a^	7.92 ± 0.89^b^
	MT 10 μM	63.50 ± 0.74^bc^	43.02 ± 1.58^a^	23.85 ± 1.32^a^	10.46 ± 0.68^a^
	MT 20 μM	65.40 ± 1.00^c^	44.31 ± 1.32^a^	20.90 ± 1.12^a^	9.40 ± 1.10^ab^
	MT 40 μM	62.56 ± 0.92^bc^	44.23 ± 1.58^a^	21.69 ± 1.39^a^	9.33 ± 0.59^ab^
	MT 80 μM	60.73 ± 1.14^b^	36.94 ± 1.46^b^	16.75 ± 1.09^b^	10.69 ± 0.82^a^
	Sig.	0.01	0.01	0.01	0.05

Six replicates were performed. Sig, significance; NS, non-significant. The different superscripts within the same column indicate significant differences.

### 3.2 Effect of MitoTEMPO on sperm velocity parameters

The different values of sperm velocity parameters under the effect of MT are presented in Table 2. MitoTEMPO incorporation in extender medium during cryopreservation modified several velocity parameters after thawing and incubation up to 3 h at 37°C. MitoTEMPO significantly decreased VAP values immediately after thawing in samples treated with 40 μM (*p* < 0.01), as well as, in all concentrations of MT compared to controls after 1 h of incubation. While, after 2 h, it decreased upon 5, 20, and 80 μM MT addition, and after 3 h incubation at 5 and 80 μM MT compared to the other experimental groups.

**Table 2:** Effect of MitoTEMPO (MT) on velocity parameters (Mean ± SEM) of cryopreserved Piedmontese bull semen after different incubation periods.

Parameter	Group	Post-thawing	1 h	2 h	3 h
VAP (μm/s)	Control	77.18 ± 0.48^ab^	65.93 ± 0.53^a^	54.53 ± 0.76^a^	48.25 ± 0.44^a^
	MT 5 μM	77.51 ± 0.30^a^	62.74 ± 0.53^b^	51.63 ± 0.88^b^	45.50 ± 0.47^b^
	MT 10 μM	76.32 ± 0.33^ab^	62.70 ± 0.54^b^	55.78 ± 0.61^a^	47.46 ± 0.46^a^
	MT 20 μM	76.07 ± 0.45^bc^	62.49 ± 0.36^b^	51.64 ± 0.64^b^	48.10 ± 0.51^a^
	MT 40 μM	75.03 ± 0.42^c^	63.83 ± 0.52^b^	54.29 ± 0.41^a^	48.34 ± 0.37^a^
	MT 80 μM	76.60 ± 0.37^ab^	60.79 ± 0.51^c^	50.99 ± 0.73^b^	50.65 ± 0.45^c^
	Sig.	0.01	0.01	0.01	0.01
VSL (μm/s)	Control	62.23 ± 0.34^ac^	55.18 ± 0.48^a^	43.33 ± 0.96^ab^	33.88 ± 0.78^ab^
	MT 5 μM	63.26 ± 0.34^b^	53.01 ± 0.47^b^	41.69 ± 0.84^bc^	32.11 ± 0.69^b^
	MT 10 μM	62.86 ± 0.29^ab^	53.01 ± 0.44^b^	44.56 ± 0.67^a^	33.86 ± 0.67^ab^
	MT 20 μM	62.64 ± 0.24^ab^	51.85 ± 0.32^b^	40.49 ± 0.69^cd^	34.07 ± 0.67^a^
	MT 40 μM	61.69 ± 0.28^c^	52.53 ± 0.47^b^	42.10 ± 0.58^bc^	33.30 ± 0.54^ab^
	MT 80 μM	62.63 ± 0.31^ab^	50.27 ± 0.42^c^	39.44 ± 0.69^d^	32.92 ± 0.70^ab^
	Sig.	0.01	0.01	0.01	0.05
VCL (μm/s)	Control	134.37 ± 0.88^a^	112.65 ± 1.00^a^	95.81 ± 1.04^a^	85.25 ± 1.17^a^
	MT 5 μM	134.29 ± 0.56^a^	105.34 ± 0.95^b^	90.22 ± 1.29^b^	81.52 ± 1.08^b^
	MT 10 μM	130.38 ± 0.64^bc^	104.92 ± 0.78^b^	97.07 ± 0.92^a^	83.60 ± 1.12^ab^
	MT 20 μM	129.45 ± 0.87^c^	105.40 ± 0.51^b^	90.74 ± 0.85^b^	85.55 ± 0.76^a^
	MT 40 μM	128.14 ± 0.81^c^	108.11 ± 0.74^c^	94.59 ± 0.69^a^	83.98 ± 1.18^ab^
	MT 80 μM	132.42 ± 0.88^ab^	103.75 ± 0.88^b^	89.92 ± 1.28^b^	85.96 ± 1.41^a^
	Sig.	0.01	0.01	0.01	0.05
ALH (μm)	Control	5.74 ± 0.03^ab^	5.44 ± 0.03^a^	5.72 ± 0.13^ab^	4.76 ± 0.43^a^
	MT 5 μM	5.82 ± 0.03^a^	5.29 ± 0.03^b^	5.44 ± 0.08^b^	5.91 ± 0.50^b^
	MT 10 μM	5.66 ± 0.03^bc^	5.35 ± 0.03^bc^	6.06 ± 0.06^ac^	5.67 ± 0.40^ab^
	MT 20 μM	5.62 ± 0.04^c^	5.29 ± 0.03^b^	5.52 ± 0.12^b^	4.98 ± 0.33^ab^
	MT 40 μM	5.69 ± 0.03^bc^	5.50 ± 0.04^a^	6.19 ± 0.15^c^	5.98 ± 0.46^b^
	MT 80 μM	5.73 ± 0.04^ab^	5.41 ± 0.04^ac^	5.47 ± 0.26^b^	5.42 ± 0.45^ab^
	Sig.	0.01	0.01	0.01	0.05
BCF (Hz)	Control	23.50 ± 0.17^a^	21.69 ± 0.15^a^	17.98 ± 0.39^a^	15.35 ± 0.49
	MT 5 μM	23.15 ± 0.11^b^	20.86 ± 0.12^b^	18.05 ± 0.37^a^	14.53 ± 0.37
	MT 10 μM	23.49 ± 0.14^a^	20.81 ± 0.11^b^	18.10 ± 0.27^a^	15.19 ± 0.29
	MT 20 μM	23.64 ± 0.14^a^	20.50 ± 0.16^bc^	17.94 ± 0.33^a^	14.91 ± 0.33
	MT 40 μM	23.54 ± 0.07^a^	20.27 ± 0.12^cd^	18.22 ± 0.23^a^	14.64 ± 0.22
	MT 80 μM	23.35 ± 0.09^ab^	19.94 ± 0.12^d^	16.79 ± 0.42^b^	14.59 ± 0.31
	Sig.	0.05	0.01	0.01	NS
STR (%)	Control	81.98 ± 0.26^a^	84.31 ± 0.19^a^	79.92 ± 0.80^abc^	70.85 ± 1.15^a^
	MT 5 μM	82.35 ± 0.16^ac^	85.13 ± 0.11^b^	81.27 ± 0.39^a^	71.69 ± 0.98^a^
	MT 10 μM	83.08 ± 0.16^b^	85.15 ± 0.18^b^	80.63 ± 0.44^ab^	72.29 ± 0.94^a^
	MT 20 μM	83.08 ± 0.30^b^	83.48 ± 0.15^c^	79.29 ± 0.50^bc^	71.69 ± 0.97^a^
	MT 40 μM	82.96 ± 0.22^bc^	83.02 ± 0.17^c^	78.58 ± 0.68^c^	69.79 ± 0.84^a^
	MT 80 μM	82.40 ± 0.21^ac^	83.13 ± 0.23^c^	78.50 ± 0.56^c^	65.88 ± 0.88^b^
	Sig.	0.01	0.01	0.01	0.01
LIN (%)	Control	48.79 ± 0.33^a^	50.71 ± 0.23^a^	46.31 ± 0.69^ab^	40.46 ± 0.65^ab^
	MT 5 μM	49.15 ± 0.23^a^	51.83 ± 0.13^b^	47.27 ± 0.47^a^	40.35 ± 0.54^ab^
	MT 10 μM	50.33 ± 0.16^b^	52.08 ± 0.16^b^	47.27 ± 0.42^a^	41.44 ± 0.53^a^
	MT 20 μM	50.54 ± 0.22^b^	50.77 ± 0.18^a^	45.90 ± 0.48^ab^	40.42 ± 0.60^ab^
	MT 40 μM	50.25 ± 0.22^b^	50.20 ± 0.14^c^	46.08 ± 0.51^ab^	40.44 ± 0.43^ab^
	MT 80 μM	49.33 ± 0.17^a^	49.94 ± 0.21^c^	45.48 ± 0.46^b^	39.04 ± 0.33^b^
	Sig.	0.01	0.01	0.01	0.05

Six replicates were performed. Sig, significance; NS, non-significant; VAP, average path velocity; VSL, straight linear velocity; VCL, curvilinear velocity; ALH, amplitude of lateral head displacement; BCF, beat cross frequency; STR, straightness ([VSL/VAP] × 100); LIN, linearity ([VSL/VCL] × 100). The different superscripts within the same column indicate the statistically significant differences.

The values of VSL increased (*p* < 0.01) directly after thawing with the MT concentration of 5 μM, while decreased (*p* < 0.01) after 1 h with all concentrations of MT and after 2 h of incubation with 80 μM MT compared to controls. After 3 h of incubation, the VSL values were increased when 20 μM MT was used compared to the concentration of 5 μM.

VCL values were decreased after thawing in samples treated with 20 and 40 μM MT and after 1 h and in all MT-treated samples compared to the controls (*p* < 0.01). While after 2 h it decreased with the 5, 20, and 80 μM concentrations compared to the other groups, and after 3 h of incubation with the 5 μM concentrations compared to the controls (*p* < 0.05).

Although ALH values decreased after thawing in samples treated with 20 μM MT compared to controls (*p* < 0.05), on the contrary, the values significantly increased in samples treated with 40 μM MT after 2 and 3 h of incubation.

BCF values decreased immediately after thawing in samples treated with 5 μM MT (*p* < 0.05), in all MT-treated samples (*p* < 0.01) after 1 h of incubation and in 80 μM MT-treated samples (*p* < 0.01) after 3 h of incubation, compared to the control group.

STR percentages increased in samples treated with 10, 20, and 40 μM MT immediately after thawing, at concentrations of 5 and 10 μM MT after 1 h of incubation, while they significantly decreased in samples treated with MT at the highest concentrations (20, 40, and 80 μM after 1 h and 40 and 80 μM after 2 h and 80 μM MT after 3 h of incubation) compared to the controls. The percentage of LIN increased (*p* < 0.01) after thawing with concentrations 10, 20, and 40 μM and after 1 h with the concentrations 5 and 10 μM, while decreased (*p* < 0.01) with the concentrations 40 and 80 μM after 1 h and with the concentrations 80 μM after 2 and 3 h of incubation compared to the controls.

### 3.3 Effect of MitoTEMPO on sperm vitality, acrosome, and plasma membrane integrities

As presented in Table 3, sperm vitality was increased in all MT-treated samples compared with controls (*p* < 0.01). In addition, the sperm acrosomal integrity was improved by 20 μM MT treatment (*p* < 0.01), whereas the plasma membrane integrity was not affected by MT treatments compared to controls.

**Table 3:** Effect of MitoTEMPO (MT) on cryopreserved Piedmontese bull semen vitality, acrosome, and plasma membrane integrities (Mean ± SEM).

Experimental groups	Sperm vitality (%)	Acrosome integrity (%)	Plasma membrane integrity (%)
Control	90.92 ± 0.87^a^	89.00 ± 0.90^a^	60.17 ± 1.75
MT 5 μM	92.92 ± 0.38^b^	90.50 ± 0.79^ab^	63.00 ± 1.78
MT 10 μM	93.17 ± 0.37^b^	90.75 ± 0.73^ab^	62.25 ± 1.80
MT 20 μM	93.08 ± 0.61^b^	91.83 ± 0.65^b^	61.50 ± 1.43
MT 40 μM	92.92 ± 0.58^b^	90.58 ± 0.85^ab^	61.50 ± 1.41
MT 80 μM	93.25 ± 0.52^b^	90.75 ± 0.84^ab^	60.92 ± 1.39

Six replicates were performed. Values with different superscripts within the same column differed significantly at *p* < 0.01.

### 3.4 Effect of MitoTEMPO on sperm apoptosis

As shown in Table 4, no statistical differences were recorded regarding the percentage of normal viable sperm and apoptotic sperm between MT-treated samples and controls, however, the group treated with 20 μM MT recorded the highest percentage of normal viable sperm and the lowest percentage of apoptotic sperm. Moreover, significant differences were recorded in the percentage of necrotic sperm between the groups treated with 20 and 40 μM MT with the lowest value recorded in the 20 μM MT samples (*p* < 0.05).

**Table 4:** Effect of MitoTEMPO (MT) on cryopreserved Piedmontese bull semen apoptosis (Mean ± SEM).

Group	Normal viable sperm (%)	Necrotic sperm (%)	Apoptotic sperm (%)
Control	47.86 ± 1.60	2.23 ± 0.02^ab^	50.16 ± 2.18
MT 5 μM	46.93 ± 1.17	2.11 ± 0.36^ab^	50.96 ± 1.10
MT 10 μM	46.01 ± 0.85	2.52 ± 0.10^ab^	51.47 ± 0.80
MT 20 μM	50.44 ± 0.95	1.94 ± 0.30^b^	47.62 ± 0.79
MT 40 μM	47.00 ± 3.02	2.72 ± 0.33^a^	50.28 ± 2.76
MT 80 μM	46.25 ± 2.94	2.60 ± 0.10^ab^	51.19 ± 3.02

Six replicates were performed. Values with different superscripts within the same column differed significantly at *p* < 0.05.

### 3.5 Effect of MitoTEMPO on sperm mitochondrial membrane potential, DNA integrity (SCSA) and ROS level

As presented in Table 5, a significant increase of sperm with high MMP was recorded if treated with 80 μM MT, compared to the other experimental groups (*p* < 0.01), while the DNA integrity was significantly (*p* < 0.01) improved in the 20 μM MT group compared to controls and the highest MT-tested concentration (80 μM). On the contrary, no significant differences have been found concerning intracellular ROS levels among the different experimental groups.

**Table 5:** Effect of MitoTEMPO (MT) on cryopreserved Piedmontese bull sperm mitochondrial membrane potential (mean ± SEM).

Groups	HMMP (%)	DNA integrity (%)	ROS (%)
Control	49.25 ± 1.08^a^	93.80 ± 0.29^bc^	22.47 ± 1.56
MT 5 μM	48.62 ± 0.75^a^	94.80 ± 0.58^ab^	21.99 ± 1.35
MT 10 μM	48.93 ± 0.87^a^	95.06 ± 0.71^ab^	22.33 ± 1.30
MT 20 μM	49.37 ± 1.10^a^	96.05 ± 0.86^a^	23.07 ± 1.72
MT 40 μM	49.62 ± 0.36^a^	94.28 ± 0.30^ab^	20.19 ± 1.82
MT 80 μM	52.52 ± 1.28^b^	92.48 ± 0.73^c^	21.10 ± 1.88

Six replicates were performed. HMMP, high mitochondrial membrane potential. Values with different superscripts within the same column differed significantly at *p* < 0.01.

### 3.6 Effect of MT-treated semen on developmental potential of bovine embryos

**Figure 8:**
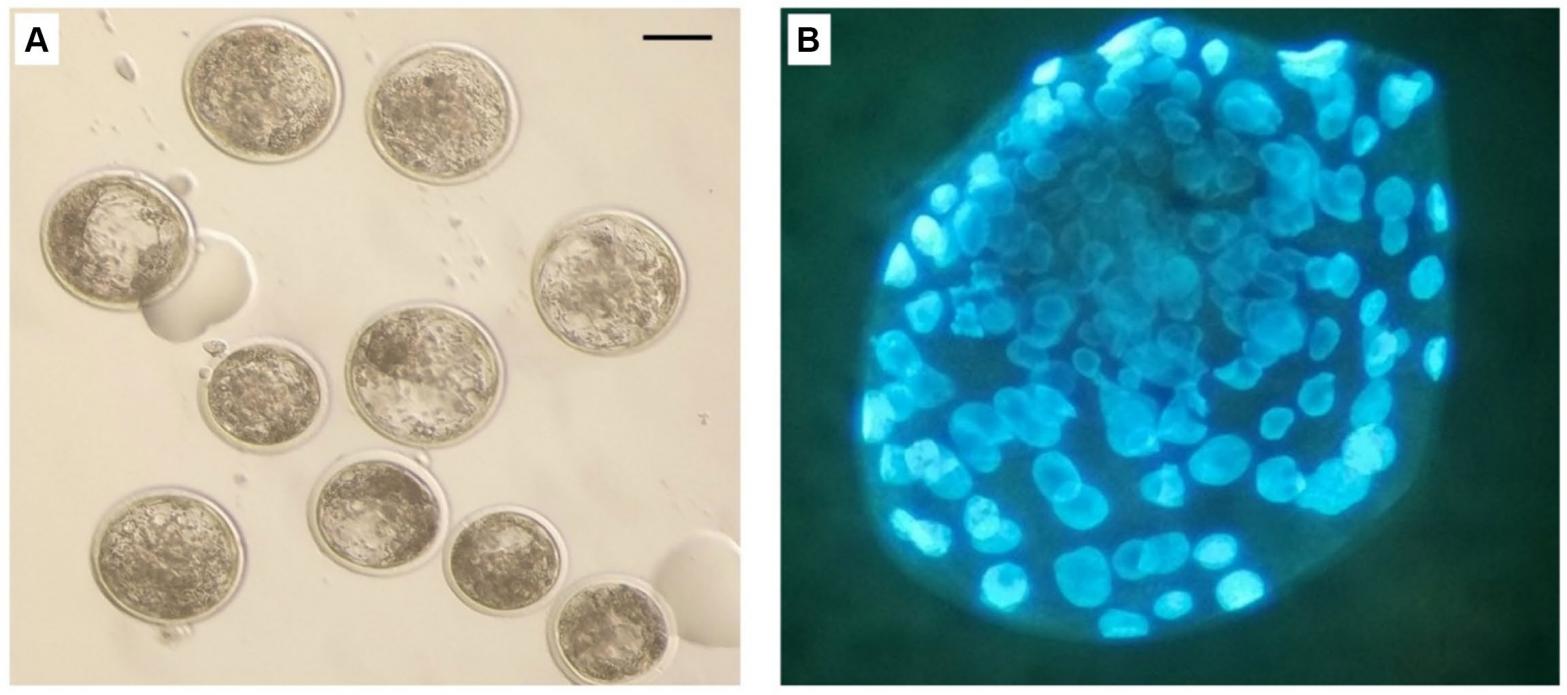
*In vitro* produced bovine blastocysts. **(A)** Normal blastocyst under stereo microscope. **(B)** blastocyst stained with Hoechst 33258. Scale bar: 100 μm.

On the basis of the results obtained on semen quality assessment, MT at a concentration of 20 μM was chosen to test the effect of MT-treated semen on the fertilizing potential of *in vitro*-produced embryos (Figure 8). A total of 193 cumulus-oocyte complexes (COCs) were cultured in six replicates (Table 6). Among these, 103 were fertilized with MT-extended semen, whereas 90 formed the control group. As shown in Table 6, the cleavage rate was significantly improved by using MT-treated spermatozoa compared with controls (46.6% vs. 38.9%; *p* < 0.05) and the blastocyst formation rates resulted in a significant increase in the MT group compared with controls (37.5% vs. 31.4%; *p* < 0.05).

**Table 6:** Effect of MitoTEMPO-treated semen at 20 μM before cryopreservation on developmental potential of *in vitro*-produced bovine embryos.

Group	Evaluated COCS	Cleavage (%)	Blastocyst (%, cleaved)
Control	90	35 (38.9)^b^	11 (31.4)^b^
MitoTEMPO 20 μM	103	48 (46.6)^a^	18 (37.5)^a^

Six replicates were performed. Chi-square test: a, b: *p* < 0.05; COC, cumulus-oocyte complex.

## 4 Discussion

Semen cryopreservation aims to prolong spermatozoa life span and maintain their fertilizing capacity (41). Nevertheless, the cryopreservation process is known to induce detrimental changes in sperm cells including membrane disruption, considerable reactive oxygen species (ROS) production, lipid peroxidation, decreased sperm motility and mitochondrial activity as well as increased damage of DNA with lowered sperm fertility either *in-vitro* or *in-vivo* (42–46). In spermatozoa, the origin of intracellular ROS is mainly mitochondrial (44, 45) and in stressful condition such as cryopreservation, sperm is exposed to oxidative damage resulting from the uncontrolled production of ROS by the cryo-injured mitochondria (47). Therefore, the use of mitochondria-targeting antioxidants (e.g., MitoTEMPO, MT) in bull semen extender could improve post-thaw sperm fertility parameters.

The sperm motility and kinetics are associated with the sperm transportation capacity to the site of fertilization and are commonly used as semen quality evaluation criteria (48, 49). Mitochondria play an important role in preserving normal sperm function and energy balance through oxidative phosphorylation and ATP synthase (50). Indeed, cryogenic mitochondrial damage has been shown to exert a detrimental effect on sperm motility through impaired ATP transport processes (51). In the present study, MT incorporation in bull semen extender improved sperm motility parameters and sperm kinetics, including STR and LIN. In agreement with our results, MT was found to improve motility and velocity parameters of frozen–thawed human (26, 27), boar (31), rooster (28), buffalo (29), and buck (30) sperm, as well as ram-chilled sperm (24). In this study, the improvement of the sperm motility, observed immediately and even up to 2 h after thawing, could be attributed to the ameliorative action of the MT in preserving sperm viability in terms of mitochondrial membrane potential, acrosome integrity and reduction of cellular necrosis.

Other studies reported that the main cause of decreased semen motility after thawing was related to the oxidative stress produced by the generation of ROS during the freezing and thawing processes (52). Precisely to overcome this limit, MT has been recently proposed as an antioxidant as it combines Tempo with triphenylphosphonium, capable of rapidly passing through the membranes of the lipid bilayer and accumulating in the mitochondria, where it is known to carry out its activity of elimination of mitochondrial superoxide anions (20, 53). In has been showed that the ROS scavenging ability of MT protected the sperm cells against oxidative stress, reduced lipid peroxidation, and maintained sperm membrane functionality and vitality (24, 26, 27). However, in the present study, MT did not reduce the intracellular ROS levels although a marked improvement in sperm quality was observed. We cannot exclude that other parameters such as lipid lipoperoxidation or the activity of specific antioxidant enzymes were influenced by MT, as demonstrated or exensively discussed in other species (24, 26–30, 54).

It has been reported that ROS and heat shocks decrease mitochondrial function, and induce mitochondrial fission, aggregation, and malfunction (51, 55, 56). Moreover, the imbalance between the production and elimination of free radicals creates oxidative stress, resulting in sperm apoptosis and DNA damage (57, 58). MitoTEMPO is a SOD mimetic antioxidant that preserves normal sperm integrity (59), and can be targeted to mitochondria for scavenging the superoxide anion and protecting against selective mitochondrial oxidant stress (60). In the present study, 20 μM MT improved DNA integrity and reduced the number of necrotic cells, however this result did not correlate with the improvement of mitochondrial activity, which was observed only after treatment with 80 μM MT.

MitoTEMPO has been reported to maintain mitochondrial function and viability during the freezing–thawing process through inhibition of mitochondrial Bax translocation (20), which is a Bcl-2 family pro-apoptotic member, that inserts into mitochondrial membranes upon cell death induction (61). In addition, MT inhibits the excessive generation and overflow of oxygen-free radicals caused by the sperm freezing–thawing process through its hydroxylamine-like structure (27). It has been stated that Glucose-6-phosphate isomerase (GPI), is an important glycolytic pathway enzyme, that loosely binds to mitochondria and is closely related to sperm quality (62). The sperm cryo-damage and stress lead to excessive release of GPI into the extracellular matrix (62, 63). MT has been reported to control the reduction of glucose-6-phosphate isomerase (GPI) activity and consequently leads to improved sperm quality (26, 28).

To our knowledge, there are no previous reports on the effect of MT on cryopreserved bull semen. In this study, MT-treated semen improved the developmental potential of bovine embryos as higher cleavage and blastocyst formation rates were obtained. Similarly, MT has been stated to improve the sperm *in vivo* fertilizing capacity of chilled (24) and frozen–thawed rooster (28) sperm. In addition, MT has been reported to improve the *in vitro* maturation and the blastocyst formation rates of bovine MT-treated oocytes (64). The improving effect of MT on the cleavage and formation rate of blastocysts could be attributed to its role in improving sperm motility and kinetic parameters which increased up to 2 h after thawing. We hypothesize this is due to the protective effect determined by MT during the cryopreservation process as it preserved cell viability in terms of acrosome and DNA integrity, reduction of cell necrosis which could explain the increase in sperm fertilizing capacity. In another study, MT has been stated to improve the quality and developmental potential of embryos through the regulation of mitochondrial functions by reducing the effect of superoxide (65). Further studies are needed to assess the fertilizing ability of sperm under the influence of MT using natural insemination and to evaluate conception and pregnancy rates.

In conclusion, the incorporation of MitoTEMPO (mainly at a concentration of 20 μM) in bull extended semen before cryopreservation has a positive protective and improving effect on different sperm parameters including motility and kinetics as well as sperm vitality, acrosomal intactness, DNA integrity, and sperm *in vitro* fertilizing capacity. Depending on these results, clinical application of MitoTEMPO could be profitable for bull frozen semen production centers.

## Data availability statement

The raw data supporting the conclusions of this article will be made available by the authors, without undue reservation.

## Ethics statement

Ethical approval was not required for the study involving animals in accordance with the local legislation and institutional requirements because no experimental animals were used in this study. All procedures were performed in accordance with DPR 27/1/1992 (Animal Protection Regulations of Italy) in compliance with European Community Regulation 86/609. Please see file “Declaration” in the section “Additional files.”

## Author contributions

AE: Conceptualization, Formal analysis, Investigation, Writing – original draft, Writing – review & editing. AR: Investigation, Writing – review & editing. AB: Investigation, Writing – review & editing. MP: Formal analysis, Investigation, Writing – review & editing. TN: Investigation, Writing – review & editing. GD: Formal analysis, Investigation, Writing – review & editing. LV: Conceptualization, Investigation, Supervision, Writing – review & editing. NM: Conceptualization, Formal analysis, Investigation, Writing – review & editing.
